# ERGA-BGE reference genome of *Holothuria (Platyperona*) *sanctori*: a sea cucumber from the Mediterranean Sea

**DOI:** 10.12688/openreseurope.20841.3

**Published:** 2026-02-03

**Authors:** Katerina Vasileiadou, Thanos Dailianis, Grigorios Skouradakis, Emmanouela Vernadou, Danae Karakasi, Astrid Böhne, Rita Monteiro, Rosa Fernández, Nuria Escudero, Tereza Manousaki, Genoscope Sequencing Team, Manon Angel, Jean-Marc Barbance, Julie Batisse, Odette Beluche, Laurie Bertrand, Elodie Brun, Maria Dubois, Corinne Dumont, Barbara Estrada, Thomas Guerin, Zineb El Hajji, Sandrine Lebled, Patricia Lenoble, Claudine Louesse, Ghislaine Magdelenat, Eric Mahieu, Claire Milani, Sophie Oztas, Marine Paillard, Emilie Payen, Emanuelle Petit, Murielle Ronsin, Benoit Vacherie, Alice Moussy, Corinne Cruaud, Karine Labadie, Lola Demirdjian, Adama Ndar, Patrick Wincker, Pedro H Oliveira, Jean-Marc Aury, Chiara Bortoluzzi

**Affiliations:** 1 Institute of Oceanography, Hellenic Centre for Marine Research, Heraklion, Crete, 70014, Greece; 2 Institute of Marine Biology Biotechnology and Aquaculture, Hellenic Centre for Marine Research, Heraklion, Crete, 70014, Greece; 3 Department of Biology, School of Sciences and Engineering, University of Crete, Vassilika Vouton, Heraklion, GR-70013, Greece; 4 Natural History Museum of Crete, School of Sciences and Engineering,, University of Crete, Knossos Avenue, Heraklion, GR-71409, Greece; 5 Leibniz Institute for the Analysis of Biodiversity Change, Adenauerallee 127, Museum Koenig Bonn, Bonn, 53113, Germany; 6 Metazoa Phylogenomics Lab, Institute for Evolutionary Biology (CSIC-UPF). Passeig marítim de la Barceloneta 37-49., Barcelona, 08003, Spain; 7 Genoscope, Institut François Jacob, CEA, CNRS, Univ Evry, Université Paris-Saclay, Evry, 91057, France; 8 Génomique Métabolique, Genoscope, Institut François Jacob, CEA, CNRS, Univ Evry, Université Paris-Saclay, Evry, 91057, France; 9 SIB Swiss Institute of Bioinformatics, Amphipôle, Quartier UNIL-Sorge, Lausanne, 1015, Switzerland

**Keywords:** Holothuria (Platyperona) sanctori, genome assembly, European Reference Genome Atlas, Biodiversity Genomics Europe, Earth Biogenome Project, Holothuriidae, sea cucumber

## Abstract

*Holothuria sanctori* is a common species of sea cucumber found in the Mediterranean Sea and the Northeast Atlantic Ocean. It typically inhabits shallow rocky and sandy seabeds, where it plays a key ecological role as a sediment engineer processing organic matter ts and thereby contributing to nutrient cycling. As an edible species, *H. sanctori* is harvested in several countries. Although it is currently listed as a species of "Least Concern" on the IUCN Red List, the absence of a regulatory framework to prevent overexploitation poses a risk of population decline. Given its ecological significance and economic value *, H. sanctori* has become a focal point in both marine conservation and aquaculture research. The entirety of the genome sequence was assembled into 23 contiguous chromosomal pseudomolecules. This chromosome-level assembly encompasses 1.2 Gb, composed of 135 contigs and 46 scaffolds, with contig and scaffold N50 values of 19.9 Mb and 50.7 Mb, respectively.

## Introduction

*Holothuria (Platyperona) sanctori* is distributed throughout the northeastern Atlantic Ocean and is particularly common in the Mediterranean Sea. This nocturnal species inhabits shallow rocky substrates at depths of up to 70 meters ( Navarro *et al.*, 2012). *Holuthuria sanctori* has a cylindrical body that is dark brown in colour, with the posterior end noticeably wider than the anterior. Its thick skin is covered with prominent dark papillae, each encircled by a whitish ring at the base. The species possesses twenty peltate tentacles and features three rows of podia and densely arranged tube feet on its ventral side ( Moussa & Wirawati, 2018). While not present in all species within the Holothuriidae family ( Caulier *et al.*, 2016), this sea cucumber is equipped with Cuvierian tubules, which serve as defensive structures. Phylogenetic analysis suggests that *H. sanctori* belongs to a deep evolutionary lineage dating back approximately 155 million years and it forms a distinct clade from other Mediterranean Holothuriidae species ( Borrero-Pérez *et al.*, 2010).

The sea cucumber *Holothuria sanctori* is a deposit feeder that primarily consumes organic matter. It ingests large amounts of sediment, extracting nutrients from bacteria, fungi, and detritus embedded within the particles. Through this activity, it plays a key ecological role as a bioturbator, contributing to sediment oxygenation and overall seabed health. *H. sanctori* is harvested in the Mediterranean region for its saponins—bioactive compounds likely produced as a chemical defence mechanism. These saponins have attracted pharmaceutical interest due to their anti-tumoral, anti-inflammatory, and antibacterial properties ( Caulier *et al.*, 2016). Additionally, the species holds significant value in aquaculture. Its ability to consume high concentrations of organic matter and filter nitrogen enables it to restore sediment quality beneath fish cages, supporting more sustainable farming practices ( Moussa & Wirawati, 2018). *Holothuria sanctori* plays a crucial role in maintaining the stability and ecological integrity of marine ecosystems. Its economic importance in both pharmaceutical and aquaculture sectors highlight the need for effective conservation strategies to ensure the sustainability of its populations in the Mediterranean.

The generation of this reference resource was coordinated by the European Reference Genome Atlas (ERGA) initiative’s Biodiversity Genomics Europe (BGE) project, supporting ERGA’s aims of promoting transnational cooperation to promote advances in the application of genomics technologies to protect and restore biodiversity ( Mazzoni *et al.*, 2023).

## Materials & methods

ERGA's sequencing strategy includes Oxford Nanopore Technology (ONT) and/or Pacific Biosciences (PacBio) for long-read sequencing, along with Hi-C sequencing for chromosomal architecture, Illumina Paired-End (PE) for polishing (i.e. recommended for ONT-only assemblies), and RNA sequencing for transcriptome profiling, to facilitate genome assembly and annotation.

### Sample and sampling information

On 13 July 2023, Katerina Vasileiadou sampled one specimen of *Holothuria sanctori* (sex unknown). The specimen was identified by Katerina Vasileiadou in Lasithi, Elounda bay, Crete, Greece based on the characteristics described by Moussa & Wirawati (2018). Sampling was performed under permission ΥΠΕΝ/ΔΔΔ/34284/1131 issued by the Ministry for Environment and Energy, Secretariat General for Natural Environment & Water, Directorate General for Forests & Forest Environment, Directorate for Forest Management, Greece.

Tissues were removed from the living specimen following the legal framework for scientific specimens, flash frozen in liquid nitrogen, and stored at -80°C until DNA extraction. The specimen was euthanized following the Greek and EU legal framework.

### Vouchering information

Physical reference materials for the here sequenced specimen have been deposited in the Natural History Museum of Crete https://www.nhmc.uoc.gr/en/ under accession number NHMC.65.25.

Frozen reference tissue material of the here sequenced specimen is available from the same individual at the Biobank of the Natural History Museum of Crete https://www.nhmc.uoc.gr/en/ under the voucher ID NHMC.65.25.

### Genetic information

The estimated genome size, based on ancestral taxa, is 2.25 Gb. This is a diploid genome with a haploid number of 21 chromosomes (2n=42). All information for this species was retrieved from Genomes on a Tree ( Challis *et al.*, 2023).

### DNA/RNA processing

DNA was extracted from 300 mg of intestinal tissue using a Genomic-tip 100/G Kit (QIAGEN, MD, USA) following manufacturer instructions. DNA fragment size selection was performed using Short Read Eliminator (PacBio, CA, USA). Quantification was performed using a Qubit dsDNA HS Assay kit (Thermo Fisher Scientific) and integrity was assessed in a FemtoPulse system (Agilent). DNA was stored at 4 °C. RNA was extracted from intestinal tissue (50 mg) using the RNeasy Plus Universal kit (Qiagen) following manufacturer instructions. Residual genomic DNA was removed with 6U of TURBO DNase (2 U/μL) (Thermo Fisher Scientific). Quantification was performed using a Qubit RNA HS Assay kit and integrity was assessed in a Bioanalyzer system (Agilent). RNA was stored at -80 °C.

### Library preparation and sequencing

Long-read DNA libraries were prepared with the SMRTbell prep kit 3.0 following manufacturers' instructions and sequenced on a Revio system (PacBio). Hi-C libraries were generated from intestinal tissue (50 mg) using the Arima High Coverage HiC kit (following the Animal Tissues low input protocol v01) and sequenced on a NovaSeq X Plus instrument (Illumina) with 2x150 bp read length. Poly(A) RNA-Seq libraries were constructed using the Illumina Stranded mRNA Prep, Ligation Prep kit (Illumina) and sequenced on an Illumina NovaSeq X Plus instrument (Illumina) with 2x150 bp read length.

In total 90x PacBio HiFi and 47x HiC data were sequenced to generate the assembly.

### Genome assembly methods

The genome of *Dendarus foraminosus* was assembled using the Genoscope GALOP pipeline ( https://workflowhub.eu/workflows/1200). Briefly, raw PacBio HiFi reads were assembled using Hifiasm v0.19.8-r603 ( Cheng *et al.*, 2021). Remaining allelic duplications were removed using purge_dups v1.2.5 ( Guan *et al.*, 2020) with default parameters and the proposed cutoffs, as well as a bacterial contig. The purged assembly was scaffolded using YaHS v1.2.2 ( Zhou *et al.*, 2023) and assembled scaffolds were then curated through manual inspection using PretextView v0.2.5 to remove false joins and incorporate sequences not automatically scaffolded into their respective locations within the chromosomal pseudomolecules. This step included the removal of a bacterial contig and a substantial number of duplications. Chromosome-scale scaffolds confirmed by Hi-C data were named in order of size. The mitochondrial genome was assembled as one circular contig using Oatk v1.0 ( Zhou *et al.*, 2024) and included in the released assembly. Summary analysis of the released assembly was performed using the ERGA-BGE Genome Report ASM Galaxy workflow ( https://doi.org/10.48546/workflowhub.workflow.1104.1).

## Results

### Genome assembly

**Figure 1.**
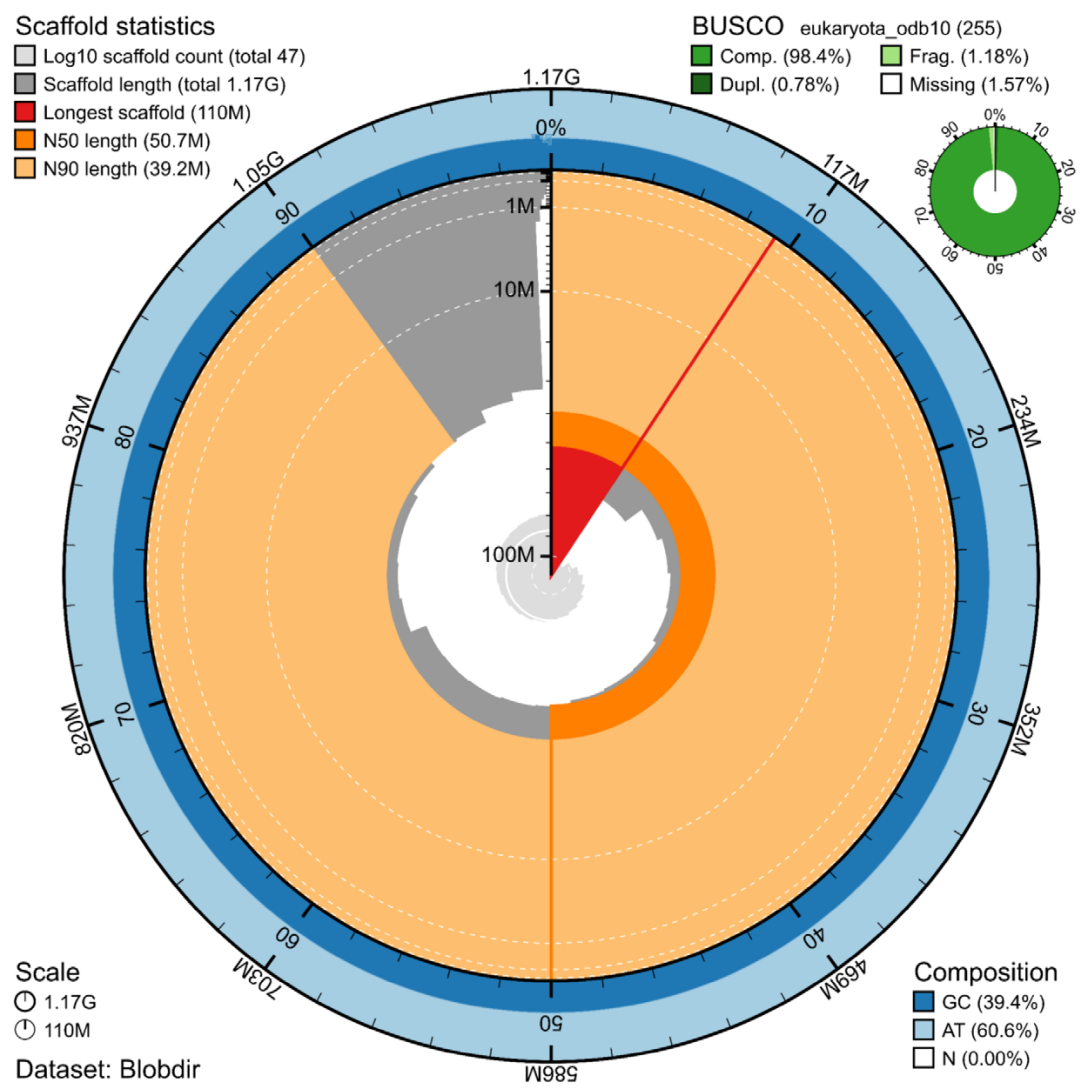
Snail plot summary of assembly statistics. The main plot is divided into 1,000 size-ordered bins around the circumference, with each bin representing 0.1% of the 1,171,681,863 bp assembly including the mitochondrial genome. The distribution of sequence lengths is shown in dark grey, with the plot radius scaled to the longest sequence present in the assembly (110 Mb, shown in red). Orange and pale-orange arcs show the scaffold N50 and N90 sequence lengths (50,699,391 and 39,181,847 bp), respectively. The pale grey spiral shows the cumulative sequence count on a log-scale, with white scale lines showing successive orders of magnitude. The blue and pale-blue area around the outside of the plot shows the distribution of GC, AT, and N percentages in the same bins as the inner plot. A summary of complete, fragmented, duplicated, and missing BUSCO genes found in the assembled genome from the Eukaryota database (odb10) is shown in the top right.

**Figure 2.**
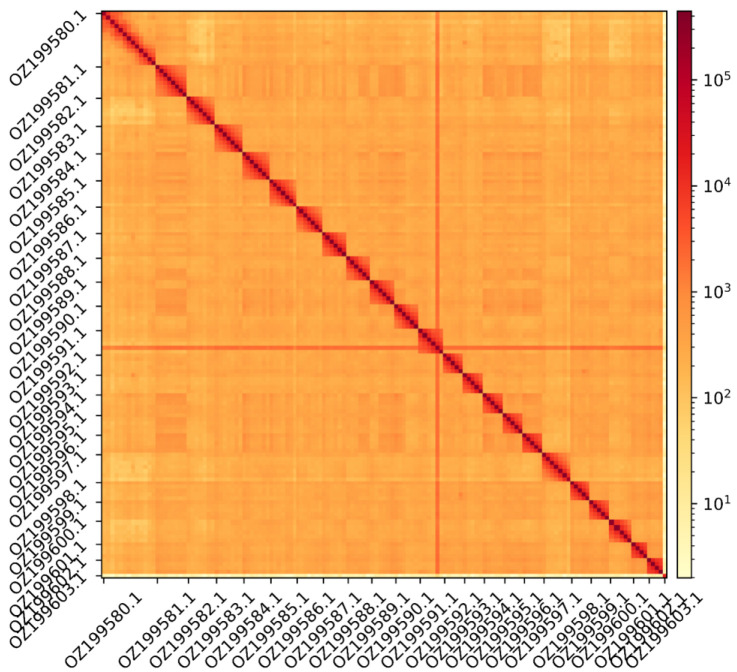
Hi-C contact map showing spatial interactions between regions of the genome. The diagonal corresponds to intra-chromosomal contacts, depicting chromosome boundaries. The frequency of contacts is shown on a logarithmic heatmap scale. Hi-C matrix bins were merged into a 150 kb bin size for plotting. The Hi-C contact map shows the 23 autosomes and the mitochondrial genome (GenBank accession: OZ199603.1).

The genome assembly has a total length of 1,171,681,863 bp in 46 scaffolds including the mitogenome ( Figure 1 & Figure 2), with a GC content of 39.4%. The assembly has a contig N50 of 19,932,592 bp and L50 of 21 and a scaffold N50 of 50,699,391 bp and L50 of 10. The assembly has a total of 89 gaps, totalling 15.0 kb in cumulative size. The single-copy gene content analysis using the Eukaryota database with BUSCO ( Manni *et al.*, 2021) resulted in 98.4% completeness (97.6% single and 0.8% duplicated). 73.3% of reads k-mers were present in the assembly and the assembly has a base accuracy Quality Value (QV) of 62.3 as calculated by Merqury ( Rhie *et al.*, 2020).

## Data Availability

*H. sanctori* and the related genomic study were assigned to Tree of Life ID (ToLID) 'ehHolSanc2' and all sample, sequence, and assembly information are available under the umbrella BioProject PRJEB77253. The sample information is available at the following BioSample accessions: SAMEA115125951 and SAMEA115125945. The genome assembly is accessible from ENA under accession number GCA_964304565.2, while the annotated genome will be made available through the Ensembl website ( https://projects.ensembl.org/erga-bge/). The genome will be annotated using the RNA-Seq data generated alongside the genome assembly (see DNA/RNA processing). Sequencing data produced as part of this project are available from ENA at the following accessions: ERX14170116, ERX14170117, ERX13549354, ERX13549355, ERX14096391, ERX14096392, and ERX14096393. Documentation related to the genome assembly and curation can be found in the ERGA Assembly Report (EAR) document available at https://github.com/ERGA-consortium/EARs/tree/main/Assembly_Reports/Holothuria_sanctori/ehHolSanc2. Further details and data about the project are hosted on the ERGA portal at https://portal.erga-biodiversity.eu/data_portal/491806.

## Author information

Members of the Genoscope Sequencing Team are listed here: https://zenodo.org/records/14611490.
