# Supplementary material for: ERGA-BGE reference genome of Holothuria (Platyperona) sanctori: a sea cucumber from the Mediterranean Sea
Source: Open Res Eur. 2026 Feb 3;5:297. Originally published 2025 Sep 25. [Version 3] doi: 10.12688/openreseurope.20841.3 (PMC12754360; doi:10.12688/openreseurope.20841.3)
Supplement: Peer review history

# Reviewer report: Reviewer response for version 1

**DOI:** 10.21956/openreseurope.22549.r64808

**Published:** 2025-12-30

**Authors:** Estelle Proux-Wéra

**Affiliations:** 1 Stockholm University, stockholm, Sweden

The authors present the genome assembly of *Holothuria (Platyperona*) *sanctori*, a sea cucumber.

 The materials and methods section describes in details how the specimen was sampled, how the DNA was extracted, and how the genome assembly was performed.

 The raw data and the final assembly are available and find able through the links provided in the article.

 The genome assembly seems to be of high quality, with an expected number of chromosomes and good metrics (N50, L50). The BUSCO score is very good.

 I was wondering about contamination, as there is nothing mentioned in the article. I could see in the EAR report that 1 bacterial contig was removed. I think it would be good to write it as well in the article.

Are sufficient details of methods and materials provided to allow replication by others?

Yes

Is the rationale for creating the dataset(s) clearly described?

Yes

Are the datasets clearly presented in a useable and accessible format?

Yes

Are the protocols appropriate and is the work technically sound?

Yes

Reviewer Expertise:

Bioinformatics, genome assembly, comparative genomics

I confirm that I have read this submission and believe that I have an appropriate level of expertise to confirm that it is of an acceptable scientific standard.

# Response

**Published:** 2026-01-05

**Authors:** Chiara Bortoluzzi

**Affiliations:** Swiss Institute of Bioinformatics, Lausanne, Vaud, Switzerland

We thank the reviewer for their comments. We have addressed all of them as following:  1. Following reviewer 1, we added to the Genome assembly methods section additional information about the removal of a bacterial contig and a substantial number of duplications during the assembly step.

# Reviewer report: Reviewer response for version 1

**DOI:** 10.21956/openreseurope.22549.r64816

**Published:** 2025-12-29

**Authors:** Poppy Hesketh-Best

**Affiliations:** 1 IGTP Institut Germans Trias i Pujol, Barcelona, Spain

In this data note, Vasileiadou and colleagues present a reference genome for *Holothuria (Platyperona) sanctori*, a sea cucumber species important for maintaining seabed health through its bioturbation activities. The reference genome consists of 46 scaffolds, including the mitochondrial genome, and represents a valuable resource for future research.

**Is the rationale for creating the dataset clearly described?**

 Yes. The rationale is clearly articulated within the broader context of the European Reference Genome Atlas initiatives. In addition, the specific justification for generating a reference genome for this sea cucumber species is well described.

**Are the protocols appropriate and is the work technically sound?**

 The protocols used for sequencing and assembling the genome are appropriate for marine invertebrates, and the technical approach for assembly is sound.

**Are sufficient details of methods and materials provided to allow replication by others?**

 Yes. The manuscript provides sufficient detail on DNA extraction, library preparation, and genome assembly to enable replication and to support appropriate downstream use of the reference genome.

 One point of confusion is the subtitle "DNA/RNA processing" in the methods. Transcriptomics is not  integrated into the results describing dataset generation. The authors could clarify the availability and relevance of the transcriptome, or remove mention of the RNA storage and focus on DNA and the genome.

 Additionally, the curator’s notes indicate the removal of a bacterial contig and a substantial number of duplications; these steps contributed to the final reference genome and should be explicitly reported in the data note.

**Are the datasets clearly presented in a usable and accessible format?**

 Yes. As of 2025-12-09, both the genome and transcriptome datasets were accessible through ENA and NCBI. The GitHub repository containing curation and assembly reports is also available and useful, although more of this information could be incorporated directly into the data note.

Are sufficient details of methods and materials provided to allow replication by others?

Yes

Is the rationale for creating the dataset(s) clearly described?

Yes

Are the datasets clearly presented in a useable and accessible format?

Yes

Are the protocols appropriate and is the work technically sound?

Yes

Reviewer Expertise:

Bioinformatics and (meta-)genomics

I confirm that I have read this submission and believe that I have an appropriate level of expertise to confirm that it is of an acceptable scientific standard.

# Response

**Published:** 2026-01-05

**Authors:** Chiara Bortoluzzi

**Affiliations:** Swiss Institute of Bioinformatics, Lausanne, Vaud, Switzerland

We thank the reviewer for their comments. We have addressed all of them as following: 

 1. We slightly changed the text of the Data availability section regarding the annotation by stating that, while the RNA-seq data are available, the annotation is not yet and will be made available in the Ensembl website. 

 2. We added in the Genome assembly methods section that a bacterial contig and  a substantial number of duplications were removed in the assembly step.
